# A Case of Distal Common Bile Duct Obstruction Due to a Neuroendocrine Tumor

**DOI:** 10.7759/cureus.52729

**Published:** 2024-01-22

**Authors:** Ramsey Rayes, Omeed Partovi, Stephanie Strohbeen, Britta L Bureau, Tamara Giorgadze, Mohamed Mostafa, Nisar Asmi

**Affiliations:** 1 Internal Medicine, Medical College of Wisconsin, Wauwatosa, USA; 2 Neurology, Medical College of Wisconsin, Wauwatosa, USA; 3 Pathology, Medical College of Wisconsin, Wauwatosa, USA; 4 Pathology, University of Arizona, Phoenix, Phoenix, USA

**Keywords:** grade 3 neuroendocrine carcinoma, case report, common bile duct neoplasm, neuroendocrine tumors (nets), common bile duct dilatation

## Abstract

Neuroendocrine tumors (NETs) are a rare subset of malignancies in the biliary tract that may have an aggressive and initially asymptomatic course. A 93-year-old female presented with four days of abdominal pain with associated nausea, jaundice, and brown-colored urine. A CT scan revealed a soft-tissue lesion measuring 1.9 x 1.5 x 1.9 cm within the distal-most aspect of the common bile duct and papilla with marked bile duct dilatation, pancreatic duct dilatation, and multiple hepatic lesions of varying sizes. The biliary stricture was palliated with a stent via endoscopic retrograde cholangiopancreatography. Biopsies taken from the biliary mass were consistent with a well-differentiated NET: World Health Organization, Grade 3. The patient was minimally symptomatic after stenting and was discharged home. She ultimately decided not to pursue further treatment and passed away two months after the initial presentation. Currently, surgical excision is considered the main and only curative treatment for localized NETs, although chemotherapy and radiation therapy may be suitable. Early detection and treatment of these rare NETs in the biliary tree can potentially result in curative treatment.

## Introduction

Neuroendocrine tumors (NETs) are a heterogeneous group of tumors that can arise from neural crest cells anywhere throughout the endocrine system with the most common locations being the gastrointestinal tract, lungs, thymus, and pancreas [1,2]. The World Health Organization (WHO) and International Agency for Research on Cancer (IARC) broadly classify neuroendocrine neoplasms into well-differentiated NETs, which are further subclassified as low, intermediate, or high-grade based on mitotic index and Ki-67 index, and poorly differentiated neuroendocrine carcinomas, which are all considered high-grade [1,2].

Within the gastrointestinal tract, the extrahepatic biliary tree mucosa lacks or is extremely scarce in enterochromaffin progenitor cells that NETs typically arise from, making NETs within these locations extremely rare [3]. Due to the subsequent lack of randomized studies and long-term follow-up, the evidence regarding the management of NETs, especially those in the biliary tree, is relatively weak compared to other types of malignancy [4]. We present a case of an elderly female who presented acutely with abdominal pain and jaundice and was found to have a NET in the distal common bile duct with metastases.

## Case presentation

A 93-year-old female, with a history of hypertension, type II diabetes, coronary artery disease, and a distant history of a duodenal ulcer, presented to the emergency department with four days of right upper quadrant (RUQ) abdominal pain and associated nausea, jaundice, and dark colored urine. She did not report vomiting, diarrhea, or notable weight loss. Prior to the presentation, the patient was in good health and lived alone.

**Figure 1:**
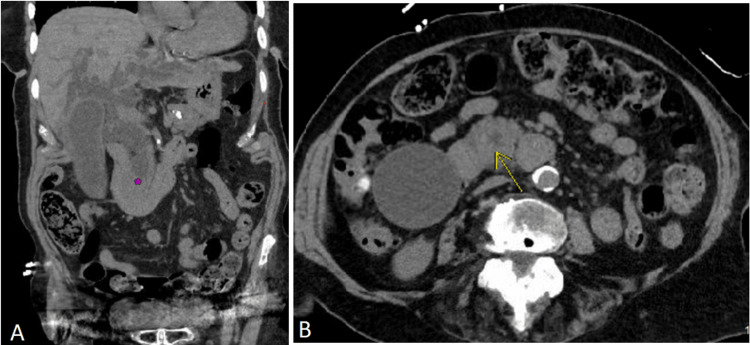
CT scan of abdomen/pelvis demonstrating a mass obstructing the common bile duct. A: Coronal CT image demonstrating a mass (purple asterisk) in the distal-most aspect of the common bile duct with marked intra- and extra-hepatic biliary duct dilatation. B: Axial CT image demonstrating a mass that is partially obstructing the distal common bile duct (yellow arrow).

Upon initial evaluation, the patient appeared jaundiced with scleral icterus and had RUQ abdominal tenderness with mild distension of the abdomen. No hepatosplenomegaly or abnormal bowel sounds were appreciated. A hepatic function panel was significant for a mixed pattern of liver injury with elevated aspartate aminotransferase (AST), alanine aminotransferase (ALT), and alkaline phosphatase (ALP) (Table 1). Of note, mildly elevated titers of ALP measuring 107-117 U/L were appreciated on multiple laboratory studies dating back 16 months prior to her presentation. Her levels of ALT and AST, however, were normal prior to this admission. CA 19-9 was markedly elevated, and her total bilirubin was increased, which was consistent with an obstructive pattern of hyperbilirubinemia (Table 1). Liver synthetic function as assessed by prothrombin time was normal. There was no leukocytosis or anemia. Lipase was within normal limits. A urine study showed elevated urobilinogen and large amounts of bilirubin. A CT scan of the abdomen and pelvis revealed a soft-tissue density measuring 1.9 x 1.5 x 1.9 cm within the distal common bile duct with marked intra- and extrahepatic bile duct dilatation, and variable pancreatic duct dilatation (Figure 1). There were multiple poorly defined hepatic lesions of varying sizes. The patient was admitted for further evaluation of a possible malignancy.

**Table 1:** Pertinent laboratory studies performed at admission (day 0) and at date of discharge (day 4; post-op day 2) with reference ranges included. Aspartate aminotransferase (AST); alanine aminotransferase (ALT); alkaline phosphatase (ALP)

Laboratory Study	Day 0 Value	Day 4 (Date of Discharge; 2 Days Post-Op) Value	Reference Range
R Factor	2.4	1.1	>5 hepatocellular; 2-5 mixed; <2 cholestatic
Aspartate aminotransferase	348	110	11-33 U/L
Alanine aminotransferase	443	208	6-37 U/L
Alkaline phosphatase	518	534	35-104 U/L
Total bilirubin	5.7	3.4	0.2-1.2 mg/dL
CA 19-9	2,694.0	-	<35.0 U/mL

Endoscopic ultrasound was subsequently done, showing a predominantly hypoechoic mass in the distal common bile duct, which measured about 20 mm and caused significant biliary dilation (27 mm). Tissue cores of the mass were then collected via fine needle aspiration (FNA). This was quickly followed by endoscopic retrograde cholangiopancreatography where a protuberant, downward-facing papilla with stricture of the common bile duct was noted. Additional intraductal biopsies were taken with forceps, and the biliary stricture was palliated with a 10 mm x 6 cm fully covered metal biliary stent. The patient tolerated the procedures well and was discharged home in stable condition two days after the procedure while biopsy results were pending.

**Figure 2:**
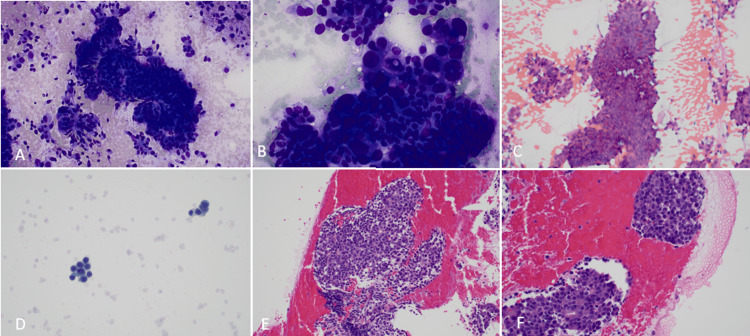
Fine needle aspiration cytology of common bile duct mass. A-B: Diff-Quick stain at 20x and 40x, respectively, showing single cells and scattered sheets of tumor cells with round to ovoid pleomorphic nuclei. Admixed are a few cells consistent with epithelial dysplasia. C: 20x Papanicolaou stain demonstrating sheets of tumor cells. D: Thin preparation nicely highlights the salt and pepper chromatin. E-F: Cell block at 20x and 40x, respectively, showing sheets and groups of cells with round to ovoid pleomorphic nuclei, salt and pepper chromatin, frequent mitotic figures, and inconspicuous nucleoli.

**Figure 3:**
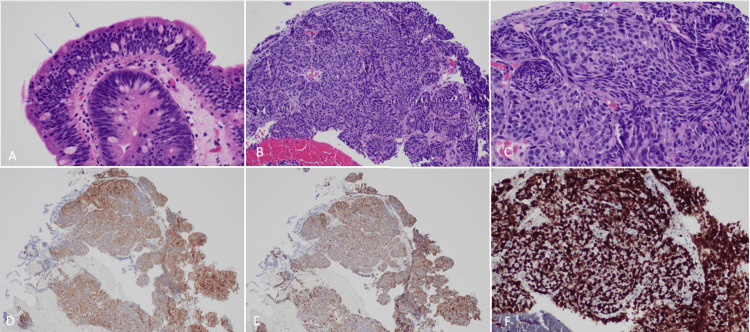
Surgical histology of common bile duct mass. A: 40x view of biliary epithelium adjacent to the tumor showing high-grade dysplasia. Note the frequent mitosis toward the luminal surface (blue arrows). B: 20x view of the neuroendocrine tumor with nested architecture. C: 40x view where neuroendocrine features can be appreciated characterized by salt and pepper chromatin. D-E: The tumor expresses synaptophysin and chromogranin immunohistochemical stains, respectively, supporting the neuroendocrine differentiation. F: MIB-1 staining shows a high Ki-67 proliferative index (>80%).

The biopsy results from the FNA demonstrated a well-differentiated neuroendocrine tumor: WHO, Grade 3 (Figure 2). On microscopic examination, there were singly scattered sheets and groups of cells with round to ovoid pleomorphic nuclei, salt and pepper chromatin, frequent mitotic figures, and inconspicuous nucleoli. Similarly, the forceps biopsy from the bile duct stricture showed sheets of neoplastic cells with oval nuclei, salt-and-pepper chromatin, and a small amount of cytoplasm (Figure 3). Immunohistochemical stains revealed the tumor cells to be positive for cytokeratin AE1/AE3, synaptophysin, chromogranin, and SSTR2A. P53 immunostain showed diffuse strong expression. MIB-1/CD45 immunostain demonstrated a Ki-67 proliferative index of 90%. The morphologic features and immunohistochemical profile from this sample were again consistent with well-differentiated neuroendocrine tumors (WHO, Grade 3).

The patient did well clinically after discharge from the hospital. Her primary complaints had been pain, fatigue, and reduced appetite although these were reportedly mild, and she had not required medication for symptom control after biliary stenting. At her follow up appointment with oncology a few weeks after discharge, she decided to forego any further testing and treatment for her condition. A periodic review of her records indicated that the patient was deceased two months after her initial presentation to the emergency department.

## Discussion

Neuroendocrine neoplasms are a heterogeneous group of tumors that can arise from neural crest cells anywhere throughout the endocrine system, with the most common locations being the gastrointestinal tract, lungs, thymus, and pancreas [1,2]. NETs are estimated to account for less than 2% of all cancers in the extrahepatic biliary tree [3-6].

The incidence, prevalence, and overall survival of NETs in general have risen over the last few decades in all age groups, likely due to earlier detection and improvement in therapies [7,8]. The annual age-adjusted incidence of NETs in the United States is estimated to be at 6.98 cases per 100,000 people with the largest increase in incidence in people aged 65 or older [7]. For extrahepatic biliary tree NETs specifically, the mean age at diagnosis has been reported to be 47.04 ± 17.62 years, with a mean diameter of 2.15 ± 1.2 cm [5]. Of these extrahepatic bile duct NETs, the most frequently documented locations involved the common hepatic duct and distal common bile duct [5].

While most NETs arise sporadically, others are associated with certain genetic conditions, such as von Hippel-Lindau disease, tuberous sclerosis, multiple endocrine neoplasia 1 and 2, and neurofibromatosis [3,9]. Further, there has been a reported increased incidence in women, which may suggest an underlying hormonal component, and Black populations [4,6-8]. For these reasons, genetic counseling and testing should be offered to patients, and appropriate family members, who have been diagnosed with an inherited disorder that increases the risk for cancer [1].

The overall progression and prognosis of extrahepatic biliary NETs are difficult to characterize given their rarity and variable presentations. It is suggested that smaller extrahepatic biliary NETs are more aggressive and tend to metastasize earlier than larger lesions [5]. While our patient had a distal common bile duct tumor diameter that was approximately equal to the reported mean by Michalopalous et al. [5], our patient already had innumerable metastatic hepatic lesions at the onset of her symptoms. Upon review of her past medical records, she had a CT scan of the chest without contrast approximately 11 months prior to her symptomatic presentation, which showed multiple low-attenuation hepatic lesions that were considered to be cysts or hemangiomas in the absence of established malignancy. Liver function panels showed mildly elevated levels of alkaline phosphatase dating back 16 months prior to her admission. It is, therefore, suggested that our patient’s malignant disease had an asymptomatic course for an extended time prior to her presentation.

While NETs have the potential to secrete various hormones, such as serotonin, vasoactive intestinal peptide, glucagon, insulin, and more, most cases of NETs in the biliary tree present with symptoms due to local invasion or metastases of the tumor [5]. Our patient presented with only abdominal pain and jaundice, which is in line with these reported findings. There have not been any documented cases of symptoms consistent with carcinoid syndrome in strictly extrahepatic biliary NETs [5]. Despite extensive metastatic disease to the liver that may have allowed vasoactive compounds to bypass hepatic metabolism, this patient did not have any complaints consistent with carcinoid syndrome.

Currently, there are no widely accepted plasma tumor markers to screen for NETs. While this case presented with an elevated CA 19-9, this tumor marker that is often used for diagnosing pancreaticobiliary malignancies can be elevated in benign biliary processes, such as obstructive jaundice [10]. CA 19-9 can provide some predictive value in prognosis for known high-grade NETs [11]. Chromogranins are glycoproteins that are stored in neuroendocrine cells and can be secreted in amounts above 10,000 pg/L in advanced NETs [12]. More specifically, a combination of chromogranins A and B have been reported to be elevated in NETs with the highest levels from tumors originating in the midgut, but these serum markers were also elevated in endocrine pancreatic tumors, pheochromocytomas, and small cell lung cancers [13].

Surgical excision is considered the main and only curative treatment for localized NETs, although chemotherapy, ablation therapies, and targeted radionuclide treatments may also be utilized depending on the disease staging [4,5]. While there is no clear role of immunotherapy for neuroendocrine neoplasms at this time, this is an area that is gaining traction [14]. The overall five-year survival rate for NETs across all stages is estimated to be 67.2% [8]. As expected, more advanced staging and grading of tumors is associated with a poorer prognosis [7,8]. While there is no explicit data regarding survival rates of extrahepatic biliary NETs, the long-term survival of these tumors appears to be significantly higher than other biliary cancers [5].

## Conclusions

We present a case of a rare extrahepatic NET to reinforce and expand prior literature to increase awareness with the goal of an earlier diagnosis of NETs. Because NETs are a rare subset of malignancies in the biliary tract that may have an aggressive and initially asymptomatic course, early detection and treatment of these tumors may result in curative treatment. Our case is unique in that there was an asymptomatic primary tumor with early metastasis to the liver. Multiple hepatic metastases were evident even before obstruction of the common bile duct became clinically evident. This emphasizes the need for early detection of these rare NETs. This case can help characterize the presentation and progression of extrahepatic biliary NETs.
